# Supplementary material for: Effects of accelerometer-based sedentary time and physical activity on DEXA-measured fat mass in 6059 children
Source: Nat Commun. 2023 Dec 12;14:8232. doi: 10.1038/s41467-023-43316-w (PMC10716139; doi:10.1038/s41467-023-43316-w)
Supplement: Supplementary file 1 — Supplementary Information [file 41467_2023_43316_MOESM1_ESM.pdf]

Effects of Accelerometer-based Sedentary Time and Physical Activity on DEXA-measured Fat Mass in 6059 Children

Andrew O. Agbaje MD, MPH[1,2], Wei Perng, MPH, PhD[3], Tomi-Pekka Tuomainen MD, PhD[1]

[1]Institute of Public Health and Clinical Nutrition, School of Medicine, Faculty of Health Sciences, University of Eastern Finland, Kuopio, Finland; [2]Children's Health and Exercise Research Centre, Department of Public Health and Sports Sciences, Faculty of Health and Life Sciences, University of Exeter, Exeter, UK; [3]Colorado School of Public Health, Lifecourse Epidemiology of Adiposity and Diabetes Center, University of Colorado Anschutz Medical Campus, Colorado, US.

Address correspondence to:
Andrew O. Agbaje, MD, MPH, Cert. Clinical Research *(Harvard)*
Institute of Public Health and Clinical Nutrition, School of Medicine, Faculty of Health Sciences, University of Eastern Finland, Kuopio Campus.
Address: Yliopistonranta 1, P.O. Box 1627, 70211 Kuopio, Finland
E-mail: andrew.agbaje@uef.fi

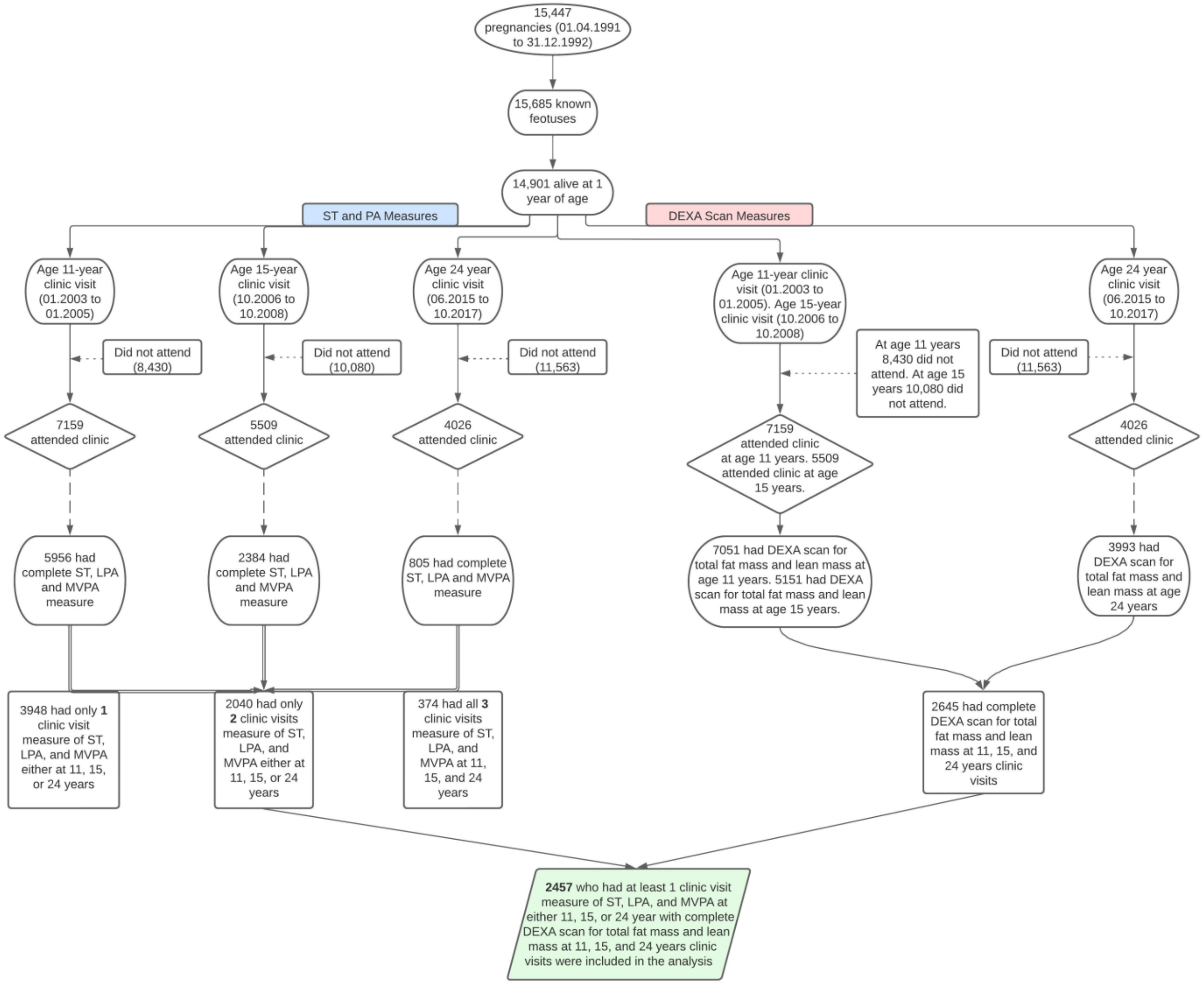

Supplemental Figure 1 Flowchart of cohort participants.

Altogether 6059 participants had at least one time-point measure of movement behaviour and body composition at either age 11, 15, or 24 years. Of 6059 participants, 917 participants had at least 2 time-point measures of movement behaviour and complete body composition measures at age 11, 15, and 24 years. Only 5217 participants attended the age 17-year clinic visits between 10.2008 and 12. 2010 of which 4953 participants had valid blood sample measures. DEXA, dual energy X-ray absorptiometry; LPA, light physical activity; MVPA, moderate to vigorous physical activity; ST, sedentary time.

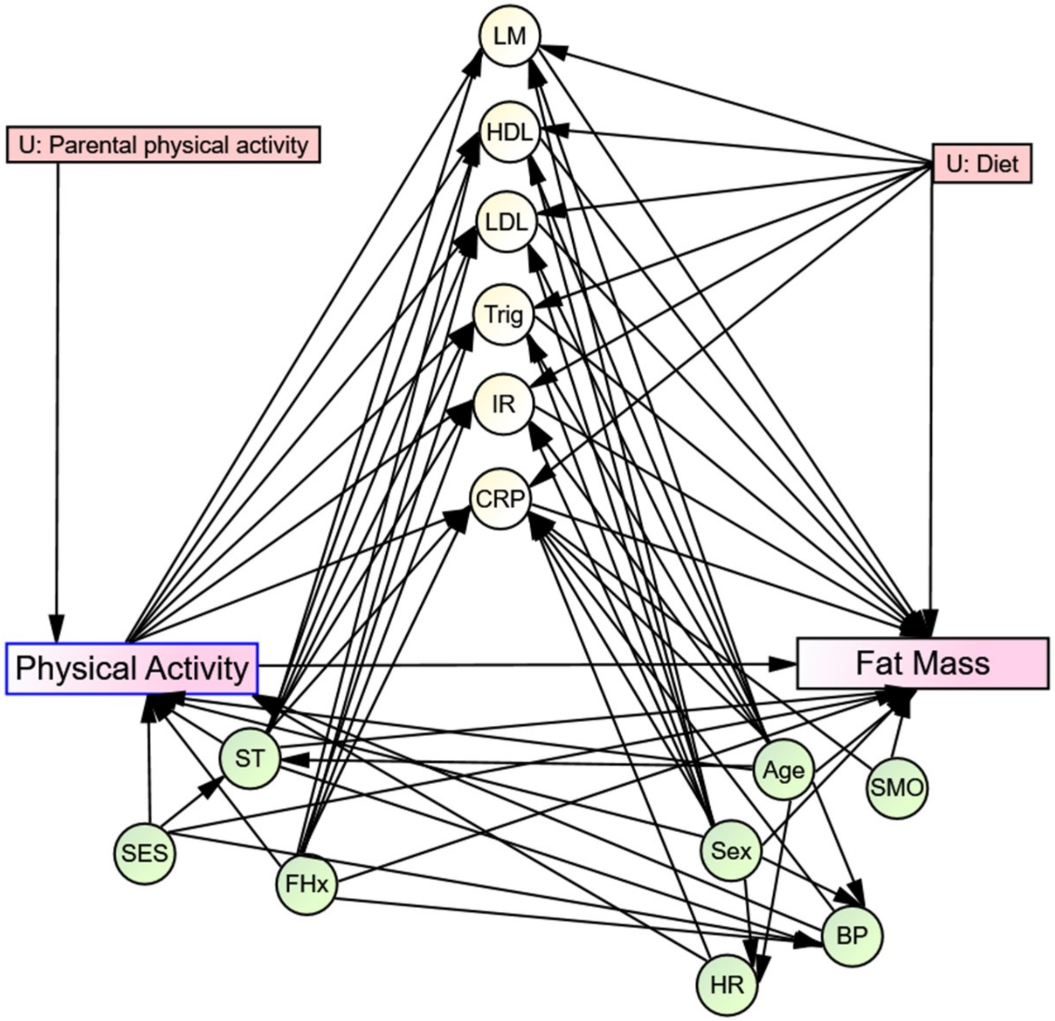

Supplemental Figure 2 Directed Acyclic Graph on the potential causal relationship between physical activity and total body fat mass, illustrating mediators (golden colour), confounders (green colour) and unmeasured confounders (red colour). BP, blood pressure; CRP, high sensitivity C-reactive protein; FHx, family history of cardiovascular, cholesterol, diabetes and hypertensive diseases; HDL, high-density lipoprotein cholesterol; HR, heart rate; IR, glucose or insulin; LDL, low-density lipoprotein cholesterol; LM, lean mass; SMO, smoking; ST, sedentary time; Trig, triglyceride; U, Unmeasured covariates.

Supplemental Table 1 Characteristics of participants excluded from the study based on **lack of at least 2 time-point measures** of movement behaviour and **incomplete** dual-energy Xray absorptiometry measure of fat mass and lean mass at either age 11-, 15-, or 24-years clinic visit using the age 15-year clinic visit profile.

Variables	**Included participants (n = 917)**	**Excluded participants (n = 1123)**		
	Mean (SD)	Mean (SD)	*P-value* for difference	Cohen's D
Age (years)	15.39 (0.22)	15.42 (0.28)	**0.018**	0.11
Anthropometry and body composition				
Body height (m)	1.68 (0.08)	1.69 (0.08)	**0.009**	0.12
*Weight (kg)	58.90 (12.4)	59.40 (13.9)	**0.025**	0.10
*Waist circumference (cm)	75.00 (9.7)	75.00 (10.5)	0.083	NA
*Body mass index (kg/m^2)	20.60 (3.82)	20.58 (4.06)	0.546	NA
*Lean mass (kg)	39.21 (11.37)	42.22 (13.01)	**<0.0001**	0.10
*Total fat mass (kg)	14.13 (10.25)	13.16 (12.73)	**0.015**	0.10
*Trunk fat mass (kg)	6.34 (5.30)	5.79 (6.28)	**0.027**	0.10
Metabolic profile				
HDL (mmol/L)	1.31 (0.29)	1.26 (0.28)	**0.002**	0.17
LDL (mmol/L)	2.09 (0.57)	2.11 (0.55)	0.850	NA
*Triglyceride (mmol/L)	0.74 (0.37)	0.73 (0.40)	0.725	NA
*C-reactive protein (mg/L)	0.34 (0.51)	0.41 (0.70)	**0.004**	0.12
*Insulin (mU/L)	8.99 (5.23)	8.83 (5.68)	0.662	NA
Glucose (mmol/L)	5.20 (0.40)	5.24 (0.36)	0.051	NA
Vascular measure				
Heart rate (beats/mins)	75 (12)	74 (13)	0.142	NA
Systolic blood pressure (mm Hg)	123 (11)	123 (10)	0.675	NA
Diastolic blood pressure (mm Hg)	67 (8)	68 (9)	**0.047**	0.09
Lifestyle factors				
Smoking status (n, %)	92 (10.12)	114 (11.01)	0.555	NA
Family history of HDCV (n, %)	250 (30.45)	221 (27.94)	0.228	NA

Altogether, 1123 participants were excluded from the study due to lack of movement behaviour variables or having a single time-point movement behaviour measure (Sedentary time, light physical activity and moderate to vigorous physical activity). The values are means (standard deviations) and *median (range/interquartile range) except for smoking status and family history in percentage. Differences between participants were tested using Student's t-test for normally distributed continuous variables, Mann–Whitney U test for skewed continuous variables, and Chi-square test for dichotomous variables. A 2-sided P-value <0.05 is considered statistically significant and is bolded. Cohen's D effect size was calculated for statistically significant differences in continuous variables: 0.2 = small, 0.5 = moderate, 0.8 = large effect. HDCV, hypertension/diabetes/high cholesterol/vascular disease; NA, not applicable; Smoking status, participants had smoked cigarettes in the past 30 days.

Supplemental Table 2 Descriptive characteristics of 917 participants who had 2 time-point measures of movement behaviour at either 11, 15 or 24 years and complete dual-energy Xray absorptiometry measure of fat mass and lean mass at age 11, 15, and 24 years clinic visits.

**Age at clinic visits/follow-up**	**11 years**			**15 years**			**24 years**		
**Variables**	**Male (n= 334)**	**Female (n= 583)**	**P-value**	**Male (n= 334)**	**Female (n= 583)**	**P-value**	**Male (n = 334)**	**Female (n= 583)**	**P-value**
Anthropometry									
Age at clinic visit (years), mean (SD)	11.71 (0.21)	11.70 (0.20)	0.757	15.39 (0.21)	15.39 (0.22)	0.724	24.49 (0.78)	24.39 (0.74)	0.058
Height (m), mean (SD)	1.50 (0.07)	1.51 (0.08)	0.532	1.75 (0.08)	1.65 (0.06)	<0.0001	1.80 (0.07)	1.66 (0.06)	<0.0001
*Weight (kg)	41.20 (11.8)	41.40 (12.2)	0.319	62.25 (13.6)	56.40 (11.2)	<0.0001	78.45 (17.42)	64.20 (17.50)	<0.0001
Body composition									
*Total fat mass (kg)	8.17 (7.69)	10.76 (7.63)	<0.0001	8.52 (7.18)	17.10 (9.02)	<0.0001	18.42 (11.70)	21.74 (12.76)	<0.0001
*Trunk fat mass (kg)	3.09 (3.56)	4.38 (3.63)	<0.0001	3.70 (3.55)	7.69 (4.76)	<0.0001	9.12 (7.05)	9.86 (6.87)	0.008
*Lean mass (kg)	29.88 (5.34)	28.42 (6.33)	<0.0001	49.55 (8.52)	36.73 (5.00)	<0.0001	56.49 (10.62)	40.81 (6.45)	<0.0001
Lean mass/fat mass ratio, mean (SD)	3.63 (2.95)	2.65 (1.62)	<0.0001	5.72 (4.83)	2.15 (1.08)	<0.0001	3.01 (1.82)	1.83 (0.85)	<0.0001
*Body mass index (kg/m^2)	17.97 (3.98)	18.16 (4.02)	0.386	20.20 (3.67)	20.80 (3.95)	0.001	24.30 (4.85)	23.21 (5.74)	0.005
*Waist circumference (cm)	66.0 (11.0)	64.70 (10.0)	0.003	74.65 (8.6)	75.10 (10.7)	0.807	84.03 (12.18)	75.0 (13.90)	<0.0001
Vascular measures									
Heart rate (beat/mins), mean (SD)	74 (10)	78 (10)	<0.0001	71 (11)	77 (12)	<0.0001	65 (11)	68 (9)	<0.0001
Systolic blood pressure (mmHg), mean (SD)	104 (9)	105 (10)	0.207	126 (11)	121 (10)	<0.0001	122 (10)	111 (9)	<0.0001
Diastolic blood pressure (mmHg), mean (SD)	59 (6)	59 (7)	0.735	68 (9)	67 (8)	0.151	67 (8)	66 (7)	0.128
Lifestyle and sociodemographic factors									
Smoked in the last 30 days (n,%)	<7 (0.3)	10 (1.8)	0.064	23 (7.0)	69 (11.9)	0.022	87 (26.3)	141 (24.3)	0.525
Family history of H-D-C-V (n,%)	90 (30.7)	160 (30.8)	0.999	NA			NA		
Sedentary time (min/day), mean (SD)	364 (69)	368 (71)	0.395	470 (83)	484 (78)	0.031	531 (84)	531 (92)	0.996
Light physical activity (min/day), mean (SD)	364 (55)	365 (57)	0.914	284 (66)	265 (62)	<0.0001	144 (46)	151 (56)	0.372
MVPA (min/day), mean (SD)	67 (42)	46 (19)	<0.0001	56 (32)	38 (21)	<0.0001	60 (36)	48 (28)	0.009
MVPA <40 mins/day (n,%)	62 (19.0)	236 (41.5)	<0.0001	93 (33.7)	267 (58.4)	<0.0001	23 (35.4)	65 (46.4)	0.107
MVPA 40 – <60 mins/day (n,%)	89 (27.2)	227 (40.0)	<0.0001	75 (27.2)	126 (27.6)	<0.0001	16 (24.6)	33 (23.6)	0.107
MVPA ≥ 60 mins/day (n,%)	176 (53.8)	105 (18.5)	<0.0001	108 (39.1)	64 (14.0)	<0.0001	26 (40)	42 (30.0)	0.107
Ethnicity- White (n,%)	300 (96.8)	526 (97.2)	0.680	NA			NA		
Maternal social economic status (n,%)			0.673	NA			NA		
Professional	14 (8.0)	20 (7.1)							
Managerial and technical	69 (39.2)	100 (35.5)							
Skilled non-manual	63 (35.8)	111 (39.4)							
Skilled manual	<6 (0.6)	6 (2.1)							
Partly skilled	21 (11.9)	35 (12.4)							
Unskilled	8 (4.5)	10 (3.5)							
Fasting plasma metabolic indices	***15 years***			***17 years***			***24 years***		
High-density lipoprotein (mmol/L), mean (SD)	1.23 (0.28)	1.36 (0.29)	<0.0001	1.19 (0.28)	1.34 (0.32)	<0.0001	1.40 (0.38)	1.65 (0.41)	<0.0001
Low-density lipoprotein (mmol/L), mean (SD)	1.99 (0.38)	2.17 (0.57)	<0.0001	2.00 (0.58)	2.21 (0.66)	<0.0001	2.49 (0.80)	2.40 (0.76)	0.146
*Triglyceride (mmol/L)	0.74 (0.32)	0.74 (0.40)	0.306	0.76 (0.35)	0.73 (0.38)	0.437	0.92 (0.59)	0.80 (0.45)	<0.0001
Glucose (mmol/L), mean (SD)	5.31 (0.38)	5.14 (0.40)	<0.0001	5.13 (0.37)	4.91 (0.36)	<0.0001	5.49 (1.06)	5.20 (0.52)	<0.0001
*Insulin (mU/L)	7.86 (4.47)	9.79 (5.29)	<0.0001	5.71 (4.17)	7.55 (4.13)	<0.0001	7.19 (5.06)	7.62 (5.71)	0.268
*High sensitivity C-reactive protein (mg/L)	0.33 (0.49)	0.35 (0.53)	0.544	0.43 (0.56)	0.56 (1.23)	0.010	0.63 (1.16)	0.93 (1.95)	<0.0001

The values are means (standard deviations) and *median (interquartile range) except for lifestyle factors and ethnicity. Differences between sexes were tested using Student's t-test for normally distributed continuous variables, Mann–Whitney U test for skewed continuous variables, Chi-square test for dichotomous variable, and analysis of covariance for multicategory variable. A 2-sided P-value <0.05 is considered statistically significant. H-D-C-V, hypertension/diabetes/high cholesterol/vascular disease; MVPA, moderate-to-vigorous physical activity; NA, not available/applicable; p-value for sex differences.

Supplemental Table 3 Longitudinal associations of cumulative sedentary time and physical activity with body composition from ages 11 through 24 years.

**N=917**	**Body mass index**		**Waist circumference**		**Trunk fat mass**	
	***β** (95% CI)*	*p-value*	***β** (95% CI)*	*p-value*	***β** (95% CI)*	*p-value*
Continuous cumulative predictor variables from ages 11 – 24 years						
**Sedentary Time (mins/day)**						
Model 1	0.247 (0.227 – 0.266)	**<0.0001**	0.181 (0.156 – 0.206)	**<0.0001**	0.232 (0.212 – 0.252)	**<0.0001**
Model 2	0.023 (0.009 – 0.036)	**<0.001**	-0.044 (-0.051 – -0.038)	**<0.0001**	0.018 (0.002 – 0.033)	**0.024**
Model 3	0.022 (0.010 – 0.035)	**<0.001**	-0.037 (-0.043 – -0.030)	**<0.0001**	0.014 (0.000 – 0.029)	**0.043**
Model 4	0.021 (0.009 – 0.034)	**<0.001**	-0.037 (-0.043 – -0.030)	**<0.0001**	0.010 (-0.004 – 0.025)	0.149
**Light Physical Activity (mins/day)**						
Model 1	-0.287 (-0.305 – -0.269)	**<0.0001**	-0.316 (-0.340 – -0.292)	**<0.0001**	-0.280 (-0.298 – -0.261)	**<0.0001**
Model 2	-0.011 (-0.025 – 0.002)	0.092	0.038 (0.031 – 0.045)	**<0.0001**	-0.022 (-0.037 – -0.006)	**0.007**
Model 3	-0.001 (-0.013 – 0.011)	0.880	0.026 (0.020 – 0.033)	**<0.001**	-0.015 (-0.029 – -0.001)	**0.037**
Model 4	-0.001 (-0.013 – 0.012)	0.932	0.027 (0.020 – 0.033)	**<0.001**	-0.014 (-0.029 – 0.000)	**0.047**
**Moderate to Vigorous Physical Activity (mins/day)**						
Model 1	-0.055 (-0.080 – -0.030)	**<0.001**	0.004 (-0.025 – 0.033)	0.793	-0.062 (-0.089 – -0.034)	**<0.001**
Model 2	-0.009 (-0.024 – 0.005)	0.205	-0.001 (-0.008 – 0.006)	0.856	-0.019 (-0.040 – 0.001)	0.068
Model 3	-0.010 (-0.024 – 0.005)	0.191	-0.006 (-0.012 – 0.001)	0.086	-0.018 (-0.038 – 0.002)	0.079
Model 4	-0.010 (-0.024 – 0.005)	0.191	-0.007 (-0.013 – 0.000)	**0.046**	-0.017 (-0.037 – 0.003)	0.103
Categorical cumulative predictor variable from ages 11 – 24 years						
**Moderate to Vigorous Physical Activity (*<40mins/day as reference*)**						
40 – <60mins/day	-0.006 (-0.039 – 0.027)	0.715	0.005 (-0.011 – 0.022)	0.530	-0.038 (-0.074 – -0.002)	**0.041**
≥60mins/day	-0.030 (-0.067 – 0.008)	0.119	-0.024 (-0.042 – -0.005)	**0.013**	-0.065 (-0.110 – -0.021)	**0.004**

**N=917**	**Total fat mass**		**Lean mass**		**Lean mass/fat mass ratio**	
	***β** (95% CI)*	*p-value*	***β** (95% CI)*	*p-value*	***β** (95% CI)*	*p-value*
Continuous cumulative predictor variables from ages 11 – 24 years						
**Sedentary Time (mins/day)**						
Model 1	0.216 (0.196 – 0.236)	**<0.0001**	0.374 (0.353 – 0.395)	**<0.0001**	-0.053 (-0.074 – -0.033)	**<0.001**
Model 2	0.023 (0.008 – 0.038)	**0.003**	0.061 (0.047 – 0.074)	**<0.0001**	0.007 (-0.011 – 0.026)	0.428
Model 3	0.018 (0.004 – 0.032)	**0.010**	0.056 (0.043 – 0.068)	**<0.0001**	0.010 (-0.007 – 0.026)	0.251
Model 4	0.017 (0.003 – 0.031)	**0.018**	0.053 (0.040 – 0.066)	**<0.001**	0.010 (-0.006 – 0.027)	0.228
**Light Physical Activity (mins/day)**						
Model 1	-0.267 (-0.286 – -0.248)	**<0.0001**	-0.400 (-0.419 – -0.382)	**<0.0001**	0.095 (0.076 – 0.115)	**<0.0001**
Model 2	-0.025 (-0.040 – -0.009)	**0.002**	-0.043 (-0.056 – -0.030)	**<0.001**	0.004 (-0.014 – 0.022)	0.647

Model 3	-0.016 (-0.030 – -0.002)	**0.026**	-0.023 (-0.035 – -0.011)	**<0.001**	0.005 (-0.011 – 0.021)	0.541
Model 4	-0.015 (-0.030 – -0.001)	**0.033**	-0.022 (-0.034 – -0.010)	**<0.001**	0.007 (-0.009 – 0.024)	0.382
**Moderate to Vigorous Physical Activity (mins/day)**						
Model 1	-0.051 (-0.077 – -0.024)	**<0.001**	-0.104 (-0.136 – -0.072)	**<0.001**	0.002 (-0.021 – 0.025)	0.881
Model 2	-0.018 (-0.039 – 0.003)	0.089	-0.030 (-0.044 – -0.016)	**<0.001**	0.004 (-0.018 – 0.026)	0.736
Model 3	-0.016 (-0.037 – 0.004)	0.113	-0.022 (-0.035 – -0.009)	**0.001**	0.006 (-0.016 – 0.029)	0.598
Model 4	-0.016 (-0.036 – 0.004)	0.121	-0.021 (-0.034 – -0.008)	**0.002**	0.006 (-0.017 – 0.028)	0.608
Categorical cumulative predictor variable from ages 11 – 24 years						
**Moderate to Vigorous Physical Activity (*<40mins/day as reference)***						
40 – <60mins/day	-0.035 (-0.070 – 0.001)	0.056	-0.012 (-0.042 – 0.019)	0.456	0.031 (-0.011 – 0.074)	0.152
≥60mins/day	-0.060 (-0.104 – -0.016)	**0.008**	-0.070 (-0.107 – -0.032)	**<0.001**	0.027 (-0.025 – 0.079)	0.309

For continuous variable analyses, model 1 was unadjusted. Model 2 was adjusted for sex, family history of hypertension/diabetes/high cholesterol/vascular disease, socioeconomic status, and other time-varying covariates measured at both baseline and follow-up such as age, low-density lipoprotein cholesterol, triglyceride, high sensitivity C-reactive protein, high-density lipoprotein cholesterol, heart rate, systolic blood pressure, glucose, insulin, smoking status, and fat mass or lean mass, depending on the outcome. Model 3 was an additional adjustment for sedentary time (LPA and MVPA model) or light physical activity (Sedentary time model). Model 4 was an additional adjustment for light physical activity (MVPA model) or moderate to vigorous physical activity (Sedentary time and LPA model). For categorical predictor variable analyses, all the above-listed covariates were adjusted for in one model. Skewed covariates were logarithmically transformed. Standardized regression coefficients ***(β)*** were computed from the generalized linear mixed-effect model for repeated measures, direct effect estimates are presented; CI, confidence interval. A 2-sided P-value <0.05 is considered statistically significant. Multiple testing was corrected with Sidak correction. Multiple imputations were used to account for missing variables. For continuous variable predictors (ST, LPA and MVPA), a 1-standard deviation change is associated with a 1-standard deviation change in the outcome For categorical variable predictor (MVPA), time spent in a category in relation to the reference is associated with 1-standard deviation change in the outcome.

Supplemental Table 4 Sex-based longitudinal associations of cumulative sedentary time and physical activity with body composition from ages 11 through 24 years in 917 participants

	**Body mass index**		**Waist circumference**		**Trunk fat mass**	
	***β** (95% CI)*	*p-value*	***β** (95% CI)*	*p-value*	***β** (95% CI)*	*p-value*
			Male (n = 334)			
ST (mins/day)	0.013 (-0.008 – 0.033)	0.228	-0.014 (-0.023 – -0.006)	**0.001**	0.002 (-0.026 – 0.030)	0.888
LPA (mins/day)	-0.006 (-0.027 – 0.015)	0.581	0.007 (-0.003 – 0.018)	0.156	-0.010 (-0.039 – 0.019)	0.519
MVPA (mins/day)	-0.003 (-0.020 – 0.015)	0.759	0.003 (-0.004 – 0.010)	0.394	-0.002 (-0.025 – 0.021)	0.864
			Female (n = 583)			
ST (mins/day)	0.026 (0.010 – 0.042)	**0.002**	-0.034 (-0.042 – -0.026)	**<0.0001**	0.019 (0.005 – 0.033)	**0.009**
LPA (mins/day)	0.004 (-0.011 – 0.018)	0.607	0.028 (0.020 – 0.036)	**<0.0001**	-0.008 (-0.020 – 0.005)	0.255
MVPA (mins/day)	-0.021 (-0.041 – 0.000)	**0.045**	0.001 (-0.009 – 0.011)	0.807	-0.028 (-0.047 – -0.009)	**0.004**
	**Total fat mass**		**Lean mass**		**Lean mass/fat mass ratio**	
			Male (n = 334)			
ST (mins/day)	0.003 (-0.026 – 0.032)	0.831	0.089 (0.063 – 0.116)	**<0.0001**	0.069 (0.034 – 0.104)	**<0.0001**
LPA (mins/day)	-0.011 (-0.040 – 0.018)	0.459	-0.053 (-0.075 – -0.030)	**<0.0001**	-0.030 (-0.066 – 0.006)	0.106
MVPA (mins/day)	-0.006 (-0.031 – 0.018)	0.611	-0.005 (-0.023 – 0.013)	0.580	0.004 (-0.024 – 0.032)	0.789
			Female (n = 583)			
ST (mins/day)	0.019 (0.006 – 0.033)	**0.005**	0.044 (0.032 – 0.055)	**<0.0001**	-0.018 (-0.033 – -0.003)	**0.016**
LPA (mins/day)	-0.007 (-0.020 – 0.005)	0.264	-0.025 (-0.037 – -0.014)	**<0.0001**	0.006 (-0.008 – 0.019)	0.388
MVPA (mins/day)	-0.027 (-0.045 – -0.009)	**0.003**	-0.007 (-0.023 – 0.010)	0.431	0.028 (0.009 – 0.048)	**0.004**

Model was adjusted for family history of hypertension/diabetes/high cholesterol/vascular disease and socioeconomic status, and time-varying covariates measured at both baseline and follow-up such as age, low-density lipoprotein cholesterol, triglyceride, high sensitivity C-reactive protein, high-density lipoprotein cholesterol, heart rate, systolic blood pressure, glucose, insulin, smoking status, and fat mass, lean mass, depending on outcome with additional adjustments for sedentary time (ST), light physical activity (LPA) or moderate to vigorous physical activity (MVPA) depending on the predictor. Skewed covariates were logarithmically transformed. Standardized regression coefficients ***(β)*** were computed from generalized linear mixed-effect model for repeated measures; CI, confidence interval. A 2-sided P-value <0.05 is considered statistically significant. Multiple testing was corrected with Sidak correction. Multiple imputations were used to account for missing variables. A 1-standard deviation change in ST, LPA, and MVPA is associated with a 1-standard deviation change in the outcome.

Supplemental Table 5 Mediating or suppressing role of cumulative fasting lipids and inflammation on the longitudinal associations of cumulative sedentary behaviour and total fat mass and lean mass from ages 11 through 24 years of 917 participants.

**Cumulative sedentary time**	**Cumulative total fat mass from ages 11 – 24 years**						
	**Total effect**		**Direct effect**		**Indirect effect**		**Mediation or Suppression (%)**
Mediators	***β (95% CI)***	***p-value***	***β (95% CI)***	***p-value***	***β (95% CI)***	***p-value***	
HDL	0.455 (0.422 – 0.485)	0.003	0.454 (0.421 – 0.482)	0.004	0.001 (-0.006 – 0.004)	0.104	0.22
LDL	0.399 (0.368 – 0.431)	0.002	0.413 (0.383 – 0.444)	0.002	-0.014 (-0.029 – -0.003)	**0.016**	**3.5**
Triglyceride	0.420 (0.388 – 0.452)	0.002	0.420 (0.391 – 0.451)	0.002	-0.001 (-0.010 – 0.010)	0.975	0.24
Lean mass	0.367 (0.330 – 0.403)	0.003	0.350 (0.313 – 0.387)	0.003	0.016 (0.010 – 0.025)	**0.002**	**4.36**
High-sensitivity CRP	0.373 (0.335 – 0.405)	0.003	0.371 (0.339 – 0.400)	0.003	0.001 (-0.022 – 0.022)	0.945	0.27
**Cumulative sedentary time**	**Cumulative lean mass from ages 11 – 24 years**						
	**Total effect**		**Direct effect**		**Indirect effect**		**Mediation or Suppression (%)**
Mediators	***β (95% CI)***	***p-value***	***β (95% CI)***	***p-value***	***β (95% CI)***	***p-value***	
HDL	0.603 (0.576 – 0.629)	0.001	0.595 (0.570 – 0.620)	0.002	0.008 (0.001 – 0.014)	**0.023**	**1.33**
LDL	0.564 (0.539 – 0.590)	0.002	0.566 (0.542 – 0.591)	0.002	-0.002 (-0.006 – 0.001)	0.177	0.35
Triglyceride	0.550 (0.524 – 0.576)	0.002	0.550 (0.525 – 0.574)	0.002	0.000 (-0.007 – 0.007)	0.931	0
Total fat mass	0.509 (0.479 – 0.542)	0.002	0.503 (0.473 – 0.534)	0.002	0.006 (-0.001 – 0.013)	0.103	1.18
High-sensitivity CRP	0.554 (0.530 – 0.581)	0.001	0.555 (0.530 – 0.581)	0.002	-0.001 (-0.005 – 0.003)	0.716	0.18

Mediation structural equation model was adjusted for sex, family history of hypertension/diabetes/high cholesterol/vascular disease, socioeconomic status, and time varying covariates measured at both baseline and follow-up such as age, high sensitivity C-reactive protein, heart rate, systolic blood pressure, glucose, insulin, smoking status, and fat mass, lean mass, light physical activity and moderate-to-vigorous physical activity, with additional adjustments for high-density lipoprotein cholesterol, low-density lipoprotein cholesterol, or triglyceride depending on the mediator. β is standardized regression co-efficient. Two-sided p-value <0.05 were considered statistically significant. When the magnitude of the longitudinal association between the predictor and outcome is increased upon inclusion of a third variable, a suppression is confirmed, However, when decreased it is mediation.

Supplemental Table 6 Mediating or suppressing role of cumulative fasting lipids and inflammation on the longitudinal associations of cumulative light physical activity with cumulative total fat mass and lean mass from ages 11 through 24 years of 917 participants.

**Cumulative light physical activity**	**Cumulative total fat mass from ages 11 – 24 years**						
	**Total effect**		**Direct effect**		**Indirect effect**		**Mediation or Suppression (%)**
Mediators	***β (95% CI)***	***p-value***	***β (95% CI)***	***p-value***	***β (95% CI)***	***p-value***	
HDL	-0.530 (-0.557 – -0.504)	0.002	-0.529 (-0.555 – -0.503)	0.002	-0.001 (-0.007 – 0.005)	0.793	0.19
LDL	-0.472 (-0.499 – -0.442)	0.002	-0.466 (-0.494 – -0.439)	0.002	-0.005 (-0.018 – 0.009)	0.505	1.06
Triglyceride	-0.472 (-0.500 – -0.446)	0.002	-0.475 (-0.501 – -0.447)	0.002	0.003 (-0.009 – 0.016)	0.651	0.64
Lean mass	-0.441 (-0.479 – -0.403)	0.002	-0.440 (-0.477 – -0.405)	0.002	-0.001 (-0.007 – 0.005)	0.788	0.23
High-sensitivity CRP	-0.500 (-0.532 – -0.468)	0.002	-0.462 (-0.489 – -0.435)	0.002	-0.038 (-0.059 – 0.017)	**0.003**	**7.6**
**Cumulative light physical activity**	**Cumulative lean mass from ages 11 – 24 years**						
	**Total effect**		**Direct effect**		**Indirect effect**		**Mediation or Suppression (%)**
Mediators	***β (95% CI)***	***p-value***	***β (95% CI)***	***p-value***	***β (95% CI)***	***p-value***	
HDL	-0.660 (-0.684 – -0.634)	0.002	-0.659 (-0.682 – -0.635)	0.002	-0.001 (-0.015 – 0.013)	0.868	0.15
LDL	-0.606 (-0.628 – -0.581)	0.002	-0.606 (-0.629 – -0.582)	0.002	0.000 (-0.001 – 0.004)	0.409	0
Triglyceride	-0.581 (-0.605 – -0.552)	0.003	-0.583 (-0.606 – -0.557)	0.003	0.002 (-0.006 – 0.011)	0.630	0.34
Total fat mass	-0.559 (-0.589 – -0.528)	0.002	-0.555 (-0.586 – -0.525)	0.002	-0.005 (-0.012 – 0.001)	0.098	0.89
High-sensitivity CRP	-0.595 (-0.617 – -0.569)	0.002	-0.593 (-0.616 – -0.567)	0.002	-0.001 (-0.005 – 0.001)	0.180	0.17

Mediation structural equation model was adjusted for sex, family history of hypertension/diabetes/high cholesterol/vascular disease, socioeconomic status, and time varying covariates measured at both baseline and follow-up such as age, high sensitivity C-reactive protein, heart rate, systolic blood pressure, glucose, insulin, smoking status, and fat mass, lean mass, sedentary time and moderate-to-vigorous physical activity, with additional adjustments for high-density lipoprotein cholesterol, low-density lipoprotein cholesterol, or triglyceride depending on the mediator. β is standardized regression co-efficient. Two-sided p-value <0.05 were considered statistically significant. When the magnitude of the longitudinal association between the predictor and outcome is increased upon inclusion of a third variable, a suppression is confirmed, However, when decreased it is mediation.

Supplemental Table 7 Mediating or suppressing role of cumulative fasting lipids and inflammation on the longitudinal associations of cumulative moderate to vigorous physical activity with cumulative total fat mass and lean mass from ages 11 through 24 years of 917 participants.

**Cumulative moderate to vigorous physical activity**	**Cumulative total fat mass from ages 11 – 24 years**						
	**Total effect**		**Direct effect**		**Indirect effect**		**Mediation or Suppression (%)**
Mediators	***β (95% CI)***	***p-value***	***β (95% CI)***	***p-value***	***β (95% CI)***	***p-value***	
HDL	-0.234 (-0.280 – -0.182)	0.006	-0.241 (-0.288 – -0.188)	0.006	0.008 (0.004 – 0.013)	**0.001**	**3.42**
LDL	-0.223 (-0.268 – -0.170)	0.006	-0.221 (-0.268 – -0.165)	0.006	-0.002 (-0.010 – 0.008)	0.724	0.90
Triglyceride	-0.234 (-0.281 – -0.179)	0.006	-0.233 (-0.279 – -0.179)	0.006	-0.001 (-0.010 – 0.006)	0.709	0.43
Lean mass	-0.254 (-0.301 – -0.198)	0.006	-0.273 (-0.324 – -0.212)	0.006	0.019 (0.010 – 0.030)	**0.003**	**7.48**
High-sensitivity CRP	-0.207 (-0.254 – -0.155)	0.005	-0.200 (-0.244 – -0.143)	0.006	-0.007 (-0.021 – 0.005)	0.251	3.38
**Cumulative moderate to vigorous physical activity**	**Cumulative lean mass from ages 11 – 24 years**						
	**Total effect**		**Direct effect**		**Indirect effect**		**Mediation or Suppression (%)**
Mediators	***β (95% CI)***	***p-value***	***β (95% CI)***	***p-value***	***β (95% CI)***	***p-value***	
HDL	0.078 (0.042 – 0.116)	0.003	0.080 (0.043 – 0.118)	0.003	-0.001 (-0.004 – 0.001)	0.195	1.28
LDL	0.088 (0.050 – 0.125)	0.003	0.089 (0.053 – 0.128)	0.003	-0.001 (-0.006 – 0.004)	0.528	1.14
Triglyceride	0.086 (0.050 – 0.125)	0.003	0.088 (0.051 – 0.126)	0.003	-0.001 (-0.009 – 0.005)	0.653	1.16
Total fat mass	0.158 (0.123 – 0.197)	0.003	0.180 (0.131 – 0.227)	0.004	-0.023 (-0.043 – -0.003)	**0.018**	**14.56**
High-sensitivity CRP	0.100 (0.062 – 0.141)	0.003	0.101 (0.065 – 0.141)	0.003	0.000 (-0.007 – 0.007)	0.916	0

Mediation structural equation model was adjusted for sex, family history of hypertension/diabetes/high cholesterol/vascular disease, socioeconomic status, and time varying covariates measured at both baseline and follow-up such as age, high sensitivity C-reactive protein, heart rate, systolic blood pressure, glucose, insulin, smoking status, and fat mass, lean mass, sedentary time and moderate-to-vigorous physical activity, with additional adjustments for high-density lipoprotein cholesterol, low-density lipoprotein cholesterol, or triglyceride depending on the mediator. β is standardized regression co-efficient. Two-sided p-value <0.05 were considered statistically significant. When the magnitude of the longitudinal association between the predictor and outcome is increased upon inclusion of a third variable, a suppression is confirmed, However, when decreased it is mediation.

Supplemental Table 8 Mediating or suppressing role of cumulative fasting lipids, glucose, insulin, lean mass, and inflammation on the longitudinal associations of moderate to vigorous physical activity with cumulative total fat mass from ages 15 through 24 years in 917 participants.

**Cumulative moderate to vigorous physical activity from ages 15 – 24 years**	**Cumulative total fat mass from ages 15 – 24 years**						
	**Total effect**		**Direct effect**		**Indirect effect**		**Mediation or Suppression (%)**
Mediators	***β (95% CI)***	***p-value***	***β (95% CI)***	***p-value***	***β (95% CI)***	***p-value***	
HDL	-0.248 (-0.275 – -0.217)	0.002	-0.256 (-0.284 – -0.224)	0.002	0.008 (0.004 – 0.013)	**0.002**	**3.23**
LDL	-0.226 (-0.252 – -0.197)	0.002	-0.197 (-0.225 – -0.165)	0.002	-0.029 (-0.037 – -0.022)	**0.002**	**12.83**
Triglyceride	-0.219 (-0.245 – -0.187)	0.002	-0.224 (-0.251 – -0.196)	0.002	0.006 (-0.002 – 0.014)	0.148	2.74
Lean mass	-0.271 (-0.300 – -0.238)	0.002	-0.281 (-0.310 – -0.248)	0.002	0.010 (0.007 – 0.014)	**0.001**	**3.69**
High-sensitivity CRP	-0.212 (-0.238 – -0.183)	0.002	-0.197 (-0.223 – -0.168)	0.002	-0.015 (-0.025 – -0.004)	**0.002**	**7.08**
Glucose	-0.254 (-0.280 – -0.222)	0.002	-0.262 (-0.289 – -0.230)	0.003	0.008 (0.005 – 0.013)	**0.001**	**3.15**
Insulin	-0.220 (-0.249 – -0.192)	0.002	-0.175 (-0.204 – -0.148)	0.002	-0.045 (-0.058 – -0.034)	**0.001**	**20.45**

Mediation structural equation model was adjusted for sex, family history of hypertension/diabetes/high cholesterol/vascular disease, socioeconomic status, and time varying covariates measured at age 15 and age 24 year clinic visits such as age, high sensitivity C-reactive protein (CRP), heart rate, systolic blood pressure, glucose, insulin, smoking status, lean mass, sedentary time, light physical activity, or moderate-to-vigorous physical activity, with additional adjustments for high-density lipoprotein cholesterol, low-density lipoprotein cholesterol, or triglyceride depending on the mediator. *β* is standardized regression co-efficient. Two-sided p-value <0.05 were considered statistically significant. When the magnitude of the longitudinal association between the predictor and outcome is increased upon inclusion of a third variable, a suppression is confirmed, However, when decreased it is mediation.

Supplemental Table 9 Complete case analysis of longitudinal associations of cumulative sedentary time and physical activity with body composition from ages 11 through 24 years of 574 of 6059 participants who had complete predictors, outcomes, and covariates at all follow-up time points.

**N=574**	**Total fat mass**		**Lean mass**		**Trunk fat mass**	
	β *(95% CI)*	*p-value*	***β*** *(95% CI)*	*p-value*	***β*** *(95% CI)*	*p-value*
**ST (mins/day)**						
Model 1	0.344 (0.315 – 0.373)	**<0.0001**	0.485 (0.451 – 0.520)	**<0.0001**	0.369 (0.340 – 0.396)	**<0.0001**
Model 2	0.023 (-0.024 – 0.070)	0.346	0.116 (0.078 – 0.154)	**<0.001**	0.019 (-0.028 – 0.066)	0.435
**LPA (mins/day)**						
Model 1	-0.393 (-0.419 – -0.367)	**<0.0001**	-0.523 (-0.554 – -0.492)	**<0.0001**	-0.416 (-0.442 – -0.390)	**<0.0001**
Model 2	-0.037 (-0.093 – 0.020)	0.202	0.039 (-0.005 – 0.082)	0.084	-0.032 (-0.088 – 0.023)	0.254
**MVPA (mins/day)**						
Model 1	-0.177 (-0.262 – -0.093)	**<0.0001**	0.044 (0.004 – 0.084)	**0.032**	-0.187 (-0.272 – -0.103)	**<0.0001**
Model 2	-0.038 (-0.077 – 0.001)	0.058	0.016 (-0.004 – 0.035)	0.113	-0.043 (-0.081 – -0.004)	**0.031**

Model 1 was unadjusted, Model 2 was adjusted for sex, family history of hypertension/diabetes/high cholesterol/vascular disease, socioeconomic status, and time-varying covariates measured at both baseline and follow-up such as age, low-density lipoprotein cholesterol, triglyceride, high sensitivity C-reactive protein, high-density lipoprotein cholesterol, heart rate, systolic blood pressure, glucose, insulin, smoking status, and fat mass, lean mass, depending on outcome with additional adjustments for sedentary time (ST), light physical activity (LPA) or moderate to vigorous physical activity (MVPA) depending on the predictor. Skewed covariates were logarithmically transformed. Standardized regression coefficients ***(β)*** were computed from generalized linear mixed-effect model for repeated measures; CI, confidence interval. A 2-sided P-value <0.05 is considered statistically significant. Multiple testing was corrected with Sidak correction. A 1-standard deviation change in ST, LPA, and MVPA is associated with a 1-standard deviation change in the outcome.

Supplemental Table 10 Characteristics of participants excluded from the study who had **at least 1 time-point measures** of movement behaviour and **incomplete** dual-energy Xray absorptiometry measure of fat mass and lean mass at either age 11, 15, or 24 years clinic visit using the age 15-year clinic visit profile.

Variables	**Included participants (n = 2457)**	**Excluded participants (n = 3905)**		
	Mean (SD)	Mean (SD)	*P-value* for difference	Cohen's D
Age (years)	15.39 (0.23)	15.48 (0.34)	**<0.001**	0.31
Sex, male (n,%)	965 (39.3)	2036 (52.1)	**<0.001**	0.13
Anthropometry and body composition				
Body height (m)	1.68 (0.08)	1.70 (0.08)	**<0.001**	0.18
*Weight (kg)	58.90 (13)	60.50 (13.8)	**<0.001**	0.12
*Waist circumference (cm)	74.80 (9.7)	75.55 (10.8)	**<0.001**	0.07
*Body mass index (kg/m^2)	20.66 (3.84)	20.72 (4.01)	0.170	NA
*Lean mass (kg)	40.04 (11.91)	42.22 (13.01)	**<0.001**	0.18
*Total fat mass (kg)	14.16 (10.97)	13.12 (12.55)	**<0.001**	0.09
*Trunk fat mass (kg)	6.31 (5.52)	5.77 (6.19)	**0.002**	0.09
Metabolic profile				
HDL (mmol/L)	1.30 (0.29)	1.27 (0.29)	**0.044**	0.07
LDL (mmol/L)	2.09 (0.56)	2.10 (0.55)	0.829	NA
*Triglyceride (mmol/L)	0.75 (0.38)	0.74 (0.41)	0.884	NA
*C-reactive protein (mg/L)	0.37 (0.64)	0.41 (0.78)	**0.003**	0.06
*Insulin (mU/L)	9.10 (5.20)	8.96 (5.48)	0.853	NA
Glucose (mmol/L)	5.21 (0.40)	5.22 (0.39)	0.238	NA
Vascular measure				
Heart rate (beats/mins)	75 (12)	74 (13)	**0.011**	0.08
Systolic blood pressure (mm Hg)	123 (11)	123 (11)	0.242	NA
Diastolic blood pressure (mm Hg)	67 (9)	68 (9)	0.328	NA
Lifestyle factors				
Smoking status (n, %)	317 (12.9)	427 (10.9)	**<0.001**	0.09
Family history of HDCV (n, %)	649 (30.3)	525 (30)	0.861	NA

Altogether, 3905 participants were excluded from the study due to lack of movement behaviour variables or having a single time-point movement behaviour measure (Sedentary time, light physical activity and moderate to vigorous physical activity). The values are means (standard deviations) and *median (range/interquartile range) except for smoking status and family history in percentage. Differences between participants were tested using Student's t-test for normally distributed continuous variables, Mann–Whitney U test for skewed continuous variables, and Chi-square test for dichotomous variables. A 2-sided P-value <0.05 is considered statistically significant and is bolded. Cohen's D effect size was calculated for statistically significant differences in continuous variables: 0.2 = small, 0.5 = moderate, 0.8 = large effect. †Phi and Cramér's V effect size was calculated for statistically significant differences in categorical variables: ≤0.2 = small, 0.2 – ≤0.6 = moderate, 0.6 = large effect. HDCV, hypertension/diabetes/high cholesterol/vascular disease; NA, not applicable; Smoking status, participants had smoked cigarettes in the past 30 days.

Supplemental Table 11 Descriptive characteristics of 2457 participants who had at least one time-point sedentary time and physical activity variable and complete 3-time point DEXA fat mass measures from age 11 through 24 years.

**Age at clinic visits/follow-up**	**11 years**			**15 years**			**24 years**		
**Variables**	**Male (n= 964)**	**Female (n= 1493)**	**P-value**	**Male (n= 964)**	**Female (n= 1493)**	**P-value**	**Male (n = 964)**	**Female (n= 1493)**	**P-value**
Anthropometry									
Age at clinic visit (years), Mean (SD)	11.71 (0.20)	11.71 (0.21)	0.464	15.38 (0.22)	15.40 (0.24)	0.061	24.52 (0.77)	24.41 (0.75)	0.001
Height (m), Mean (SD)	1.51 (0.07)	1.51 (0.07)	0.111	1.75 (0.07)	1.65 (0.06)	<0.0001	1.80 (0.07)	1.66 (0.06)	<0.0001
*Weight (kg)	41 (12)	42.2 (12.6)	0.005	62.15 (13.3)	56.7 (11.6)	<0.0001	78.2 (18.1)	64.5 (16.3)	<0.0001
Ethnicity- White (n,%)	866 (96.5)	1335 (96.2)	0.733	NA			NA		
Body composition									
*Total fat mass (kg)	8.40 (8.0)	10.92 (7.97)	<0.0001	8.36 (7.88)	16.91 (9.01)	<0.0001	18.29 (11.53)	21.95 (11.71)	<0.0001
*Trunk fat mass (kg)	3.15 (3.56)	4.46 (3.85)	<0.0001	3.69 (4.0)	7.58 (4.69)	<0.0001	9.01 (6.91)	9.92 (6.68)	<0.0001
*Lean mass (kg)	29.88 (5.34)	28.66 (6.34)	<0.0001	49.75 (7.96)	36.73 (5.14)	<0.0001	56.23 (10.28)	40.70 (6.59)	<0.0001
Lean mass/fat mass ratio Mean (SD)	3.99 (2.24)	2.87 (1.31)	<0.0001	6.33 (3.45)	2.33 (0.92)	<0.0001	3.23 (1.32)	1.89 (0.64)	<0.0001
*Body mass index (kg/m^2)	17.98 (3.98)	18.42 (4)	0.006	20.27 (3.66)	20.96 (3.91)	<0.0001	24.20 (4.91)	23.35 (5.54)	0.011
*Waist circumference (cm)	66 (11.5)	74.6 (10.6)	<0.0001	74.8 (8.6)	74.6 (10.6)	0.326	83.5 (12.9)	75 (13.36)	<0.0001
Vascular measures									
Heart rate (beat/mins), Mean (SD)	74 (11)	78 (11)	<0.0001	72 (12)	77 (12)	<0.0001	65 (11)	68 (10)	<0.0001
Systolic blood pressure (mmHg), Mean (SD)	105 (9)	106 (10)	0.026	126 (10)	121 (11)	<0.0001	123 (11)	112 (9)	<0.0001
Diastolic blood pressure (mmHg), Mean (SD)	58 (6)	59 (6)	0.044	68 (9)	67 (8)	<0.0001	68 (8)	66 (8)	<0.0001
Lifestyle factors									
Smoked in the last 30 days (n,%)	<7 (0.4)	31 (2.2)	<0.0001	84 (9)	235 (15.9)	<0.0001	264 (27.8)	383 (25.8)	0.301
Family history of H-D-C-V (n,%)	247 (29.8)	403 (30.6)	0.700	NA			NA		
Sedentary time (min/day), Mean (SD)	359 (70)	366 (70)	0.017	467 (84)	485 (79)	<0.0001	528 (83)	525 (86)	0.747
Light physical activity (min/day), Mean (SD)	365 (60)	365 (58)	0.843	287 (68)	270 (63)	<0.0001	146 (58)	150 (54)	0.412
MVPA (min/day), Mean (SD)	66 (37)	46 (20)	<0.0001	54 (30)	40 (22)	<0.0001	54 (33)	48 (28)	0.038
MVPA <40 mins/day (n,%)	165 (18)	602 (43)	<0.0001	182 (35)	488 (57)	<0.0001	89 (40)	183 (45)	0.178
MVPA 40 – <60 mins/day (n,%)	264 (29)	494 (35)	<0.0001	152 (29)	232 (27)	<0.0001	60 (27)	106 (26)	0.178
MVPA ≥ 60 mins/day (n,%)	488 (53)	304 (22)	<0.0001	192 (37)	142 (17)	<0.0001	74 (33)	117 (29)	0.178
Maternal social economic status (n,%)			0.223	NA			NA		
Professional	49 (10.2)	39 (5.6)							
Managerial and technical	191 (39.9)	273 (39.2)							
Skilled non-manual	156 (32.6)	264 (37.9)							
Skilled manual	6 (1.3)	15 (2.2)							
Partly skilled	63 (13.2)	89 (12.8)							
Unskilled	14 (2.9)	17 (2.4)							
Fasting plasma metabolic indices	***15 years***			***17 years***			***24 years***		
High-density lipoprotein (mmol/L), Mean (SD)	1.22 (0.27)	1.36 (0.29)	<0.0001	1.19 (0.26)	1.36 (0.32)	<0.0001	1.40 (0.37)	1.66 (0.42)	<0.0001
Low-density lipoprotein (mmol/L), Mean (SD)	1.99 (0.53)	2.16 (0.57)	<0.0001	2.02 (0.58)	2.21 (0.63)	<0.0001	2.48 (0.78)	2.42 (0.76)	0.063
*Triglyceride (mmol/L)	0.73 (0.35)	0.76 (0.39)	0.001	0.74 (0.33)	0.74 (0.37)	0.687	0.89 (0.58)	0.80 (0.44)	<0.0001
Glucose (mmol/L), Mean (SD)	5.30 (0.43)	5.14 (0.38)	<0.0001	5.14 (0.43)	4.91 (0.36)	<0.0001	5.47 (0.83)	5.20 (0.50)	<0.0001
*Insulin (mU/L)	8.10 (4.90)	9.78 (5.43)	<0.0001	5.89 (3.71)	7.40 (4.31)	<0.0001	7.04 (5.12)	7.75 (5.63)	<0.0001
*High sensitivity C-reactive protein (mg/L)	0.38 (0.64)	0.37 (0.62)	0.103	0.43 (0.64)	0.62 (1.29)	<0.0001	1.04 (1.10)	1.01 (2.06)	<0.0001

The values are means (standard deviations) and *median (interquartile range) except for lifestyle factors and ethnicity. Differences between sexes were tested using Student's t-test for normally distributed continuous variables, Mann–Whitney U test for skewed continuous variables, Chi-square test for dichotomous variable, and analysis of covariance for multicategory variable. A 2-sided P-value <0.05 is considered statistically significant. HDCV, hypertension/diabetes/high cholesterol/vascular disease; MVPA, moderate-to-vigorous physical activity; NA, not available/applicable; p-value for sex differences.

Supplemental Table 12 Mediating or suppressing role of cumulative fasting lipids, glucose, insulin, and inflammation at 15, 17 and 24 years and lean mass at 11, 15 and 24 years on the longitudinal associations of moderate to vigorous physical activity with cumulative total fat mass during ages 11 through 24 years in 2457 participants.

**N = 2457**	**Cumulative total fat mass from ages 11 – 24 years**						
**Cumulative sedentary time from ages 11 – 24 years**	**Total effect**		**Direct effect**		**Indirect effect**		**Mediation or Suppression (%)**
Mediators	***β (95% CI)***	***p-value***	***β (95% CI)***	***p-value***	***β (95% CI)***	***p-value***	
HDL	0.536 (0.518 – 0.553)	0.002	0.536 (0.519 – 0.553)	0.002	0.000 (-0.001 – 0.001)	0.523	0
LDL	0.506 (0.488 – 0.523)	0.001	0.502 (0.485 – 0.519)	0.001	0.004 (-0.001 – 0.009)	0.122	0.79
Triglyceride	0.510 (0.494 – 0.528)	0.001	0.512 (0.497 – 0.530)	0.001	-0.002 (-0.007 – 0.002)	0.315	0.39
Lean mass	0.483 (0.461 – 0.504)	0.002	0.472 (0.450 – 0.494)	0.002	0.011 (0.008 – 0.014)	**0.001**	**2.28**
High-sensitivity CRP	0.487 (0.468 – 0.507)	0.002	0.468 (0.451 – 0.485)	0.001	0.019 (0.010 – 0.029)	**0.002**	**3.90**
Glucose	0.534 (0.518 – 0.551)	0.001	0.535 (0.519 – 0.551)	0.001	0.000 (-0.002 – 0.000)	0.158	0
Insulin	0.538 (0.520 – 0.555)	0.002	0.534 (0.519 – 0.549)	0.002	0.004 (-0.007 – 0.012)	0.462	0.74
**Cumulative light physical activity from ages 11 – 24 years**							
HDL	-0.530 (-0.548 – -0.513)	0.001	-0.529 (-0.547 – -0.513)	0.001	-0.002 (-0.004 – 0.000)	0.090	0.38
LDL	-0.480 (-0.500 – -0.464)	0.001	-0.479 (-0.497 – -0.464)	0.001	-0.002 (-0.008 – 0.005)	0.633	0.42
Triglyceride	-0.489 (-0.507 – -0.474)	0.001	-0.490 (-0.508 – -0.476)	0.001	0.001 (-0.005 – 0.007)	0.834	0.21
Lean mass	-0.447 (-0.471 – -0.422)	0.002	-0.451 (-0.474 – -0.428)	0.002	0.004 (0.001 – 0.008)	**0.014**	**0.90**
High-sensitivity CRP	-0.463 (-0.485 – -0.443)	0.001	-0.446 (-0.464 – -0.430)	0.001	-0.017 (-0.030 – -0.004)	**0.014**	**3.67**
Glucose	-0.517 (-0.535 – -0.502)	0.001	-0.516 (-0.533 – -0.501)	0.001	-0.001 (-0.003 – 0.000)	**0.040**	**0.19**
Insulin	-0.524 (-0.545 – -0.506)	0.001	-0.525 (-0.541 – -0.510)	0.001	0.000 (-0.014 – 0.014)	0.990	0
**Cumulative moderate to vigorous physical activity from ages 11 – 24 years**							
HDL	-0.291 (-0.318 – -0.261)	0.002	-0.301 (-0.327 – -0.270)	0.002	0.010 (0.007 – 0.013)	**0.002**	**3.44**
LDL	-0.276 (-0.303 – -0.246)	0.003	-0.270 (-0.298– -0.239)	0.002	-0.007 (-0.012 – -0.001)	**0.020**	**2.54**
Triglyceride	-0.281 (-0.308 – -0.250)	0.003	-0.284 (-0.311 – -0.254)	0.003	0.003 (-0.001 – 0.007)	0.179	1.07
Lean mass	-0.304 (-0.331 – -0.272)	0.001	-0.313 (-0.340 – -0.280)	0.003	0.010 (0.004 – 0.015)	**0.002**	**3.29**
High-sensitivity CRP	-0.254 (-0.281 – -0.225)	0.002	-0.246 (-0.273 – -0.215)	0.003	-0.008 (-0.015 – 0.000)	**0.045**	**3.15**
Glucose	-0.303 (-0.330 – -0.272)	0.002	-0.312 (-0.339 – -0.278)	0.003	0.009 (0.006 – 0.013)	**0.002**	**2.97**
Insulin	-0.284 (-0.331 – -0.250)	0.002	-0.263 (-0.289 – -0.232)	0.003	-0.021 (-0.026 – -0.016)	**0.001**	**7.39**

Mediation structural equation model was adjusted for sex, family history of hypertension/diabetes/high cholesterol/vascular disease, socioeconomic status, and time varying covariates measured at both baseline and follow-up such as age, high sensitivity C-reactive protein (CRP), heart rate, systolic blood pressure, glucose, insulin, smoking status, lean mass, sedentary time, light physical activity, or moderate-to-vigorous physical activity, with additional adjustments for high-density lipoprotein cholesterol, low-density lipoprotein cholesterol, or triglyceride depending on the mediator. *β* is standardized regression co-efficient. Two sided p-value <0.05 were considered statistically significant. When the magnitude of the longitudinal association between the predictor and outcome is increased upon inclusion of a third variable, a suppression is confirmed, However, when decreased it is mediation.

Supplemental Table 13 Mediating or suppressing role of cumulative fasting lipids, glucose, insulin, lean mass, and inflammation on the longitudinal associations of moderate to vigorous physical activity with cumulative total fat mass from ages 15 through 24 years in 2457 participants

**Cumulative moderate to vigorous physical activity from ages 15 – 24 years**	**Cumulative total fat mass from ages 15 – 24 years**						
	**Total effect**		**Direct effect**		**Indirect effect**		**Mediation or Suppression (%)**
Mediators	***β (95% CI)***	***p-value***	***β (95% CI)***	***p-value***	***β (95% CI)***	***p-value***	
HDL	-0.263 (-0.289 – -0.232)	0.002	-0.276 (-0.303 – -0.244)	0.002	0.013 (0.008 – 0.018)	**0.003**	**4.94**
LDL	-0.242 (-0.268 – -0.213)	0.002	-0.215 (-0.242 – -0.182)	0.002	-0.027 (-0.035 – -0.020)	**0.002**	**11.15**
Triglyceride	-0.231 (-0.258 – -0.200)	0.002	-0.236 (-0.263 – -0.207)	0.002	0.005 (-0.002 – 0.012)	0.138	2.16
Lean mass	-0.285 (-0.314 – -0.253)	0.002	-0.295 (-0.325 – -0.263)	0.002	0.010 (0.007 – 0.014)	**0.001**	**3.51**
High-sensitivity CRP	-0.225 (-0.252 – -0.195)	0.002	-0.210 (-0.238 – -0.182)	0.002	-0.014 (-0.024 – -0.003)	**0.002**	**5.49**
Glucose	-0.267 (-0.293 – -0.236)	0.002	-0.273 (-0.300 – -0.242)	0.002	0.005 (0.003 – 0.009)	**0.001**	**1.87**
Insulin	-0.214 (-0.243 – -0.184)	0.002	-0.185 (-0.214 – -0.155)	0.002	-0.029 (-0.038 – -0.021)	**0.002**	**13.55**

Mediation structural equation model was adjusted for sex, family history of hypertension/diabetes/high cholesterol/vascular disease, socioeconomic status, and time varying covariates measured at age 15 and age 24 year clinic visits such as age, high sensitivity C-reactive protein (CRP), heart rate, systolic blood pressure, glucose, insulin, smoking status, lean mass, sedentary time, light physical activity, or moderate-to-vigorous physical activity, with additional adjustments for high-density lipoprotein cholesterol, low-density lipoprotein cholesterol, or triglyceride depending on the mediator. β is standardized regression co-efficient. Tow sided p-value <0.05 were considered statistically significant. When the magnitude of the longitudinal association between the predictor and outcome is increased upon inclusion of a third variable, a suppression is confirmed, However, when decreased it is mediation.

Supplemental Table 14 Compositional data analysis of the longitudinal associations of sedentary time and physical activity with body composition from ages 11 through 24 years in 6059 participants

**N=6059**	**Total fat mass**		**Lean mass**		**Trunk fat mass**	
	β *(95% CI)*	*p-value*	***β*** *(95% CI)*	*p-value*	***β*** *(95% CI)*	*p-value*
**ST relative to LPA and MVPA**						
Model 1	0.006 (0.005 – 0.007)	**<0.0001**	0.008 (0.007 – 0.009)	**<0.0001**	0.006 (0.005 – 0.007)	**<0.0001**
Model 2	0.002 (0.001 – 0.003)	**<0.001**	0.001 (0.000 – 0.001)	0.200	0.001 (0.001 – 0.002)	**0.002**
**LPA relative to ST and MVPA**						
Model 1	-0.004 (-0.005 – -0.003)	**<0.001**	-0.006 (-0.007 – -0.005)	**<0.0001**	-0.004 (-0.005 – -0.003)	**<0.001**
Model 2	-0.001 (-0.002 – 0.000)	**0.026**	-0.001 (-0.001 – 0.000)	0.115	-0.001 (-0.001 – 0.000)	0.244
**MVPA relative to ST and LPA**						
Model 1	-0.055 (-0.065 – -0.045)	**<0.0001**	-0.061 (-0.073 – -0.049)	**<0.0001**	-0.061 (-0.072 – -0.051)	**<0.0001**
Model 2	-0.033 (-0.041 – -0.025)	**<0.001**	0.003 (-0.004 – 0.010)	0.393	-0.026 (-0.034 – -0.018)	**<0.001**
**LPA relative to ST**						
Model 1	-0.004 (-0.005 – -0.003)	**<0.0001**	-0.006 (-0.007 – -0.005)	**<0.0001**	-0.004 (-0.005 – -0.003)	**<0.0001**
Model 2	-0.001 (-0.002 – -0.001)	**<0.001**	-0.001 (-0.001 – 0.000)	0.149	-0.001 (-0.002 – 0.000)	**0.028**
**MVPA relative to ST**						
Model 1	-0.012 (-0.014 – -0.010)	**<0.0001**	-0.016 (-0.018 – -0.014)	**<0.0001**	-0.012 (-0.014 – -0.011)	**<0.0001**
Model 2	-0.005 (-0.007 – -0.004)	**<0.001**	-0.001 (-0.002 – 0.001)	0.356	-0.004 (-0.005 – -0.002)	**<0.001**

Model 1 was unadjusted, Model 2 was adjusted for sex, family history of hypertension/diabetes/high cholesterol/vascular disease, socioeconomic status, and time-varying covariates measured at both baseline and follow-up such as age, low-density lipoprotein cholesterol, triglyceride, high sensitivity C-reactive protein, high-density lipoprotein cholesterol, heart rate, systolic blood pressure, glucose, insulin, smoking status, in addition to lean mass or total fat mass depending on the outcome. Skewed covariates were logarithmically transformed. Standardized regression coefficients ***(β)*** were computed from generalized linear mixed-effect model for repeated measures; CI, confidence interval; LPA, light physical activity; MVPA, moderate to vigorous physical activity; ST, sedentary time. A 2-sided P-value <0.05 is considered statistically significant. Multiple testing was corrected with Sidak correction. Isometric logarithmic transformation of movement behaviours was used in the compositional data analysis.
