# Supplementary material for: Effects of accelerometer-based sedentary time and physical activity on DEXA-measured fat mass in 6059 children
Source: Nat Commun. 2023 Dec 12;14:8232. doi: 10.1038/s41467-023-43316-w (PMC10716139; doi:10.1038/s41467-023-43316-w)
Supplement: Supplementary file 3 — Reporting Summary [file 41467_2023_43316_MOESM3_ESM.pdf]

nature portfolio

Corresponding author(s): Andrew Agbaje

Last updated by author(s): Sep 30, 2023

Reporting Summary

Nature Portfolio wishes to improve the reproducibility of the work that we publish. This form provides structure for consistency and transparency in reporting. For further information on Nature Portfolio policies, see our Editorial Policies and the Editorial Policy Checklist.

Statistics

For all statistical analyses, confirm that the following items are present in the figure legend, table legend, main text, or Methods section.

n/a	Confirmed	
☐	☒	The exact sample size (*n*) for each experimental group/condition, given as a discrete number and unit of measurement
☐	☒	A statement on whether measurements were taken from distinct samples or whether the same sample was measured repeatedly
☐	☒	The statistical test(s) used AND whether they are one- or two-sided<br>*Only common tests should be described solely by name; describe more complex techniques in the Methods section.*
☐	☒	A description of all covariates tested
☐	☒	A description of any assumptions or corrections, such as tests of normality and adjustment for multiple comparisons
☐	☒	A full description of the statistical parameters including central tendency (e.g. means) or other basic estimates (e.g. regression coefficient) AND variation (e.g. standard deviation) or associated estimates of uncertainty (e.g. confidence intervals)
☐	☒	For null hypothesis testing, the test statistic (e.g. *F*, *t*, *r*) with confidence intervals, effect sizes, degrees of freedom and *P* value noted<br>*Give P values as exact values whenever suitable.*
☒	☐	For Bayesian analysis, information on the choice of priors and Markov chain Monte Carlo settings
☐	☒	For hierarchical and complex designs, identification of the appropriate level for tests and full reporting of outcomes
☐	☒	Estimates of effect sizes (e.g. Cohen's *d*, Pearson's *r*), indicating how they were calculated

Our web collection on statistics for biologists contains articles on many of the points above.

Software and code

Policy information about availability of computer code

Data collection	Study data at 24 years of age were collected and managed using REDCap electronic data capture tools hosted at the University of Bristol, UK. Previous data collection at ages 11, 15 and 17 years clinic visit were not managed by REDCap.
Data analysis	All statistical analyses were performed using SPSS statistics software, Version 27.0 (IBM Corp, Armonk, NY, USA), and mediation analyses and auto-regressive cross-lagged temporal causal path structural equation modeling were conducted using IBM AMOS version 27.0. This statistical software are publicly available.

For manuscripts utilizing custom algorithms or software that are central to the research but not yet described in published literature, software must be made available to editors and reviewers. We strongly encourage code deposition in a community repository (e.g. GitHub). See the Nature Portfolio guidelines for submitting code & software for further information.

Data

Policy information about availability of data

All manuscripts must include a data availability statement. This statement should provide the following information, where applicable:

- Accession codes, unique identifiers, or web links for publicly available datasets
- A description of any restrictions on data availability
- For clinical datasets or third party data, please ensure that the statement adheres to our policy

The informed consent obtained from ALSPAC participants does not allow the data to be made freely available through any third-party maintained public repository. However, data used for this submission can be made available on request to the ALSPAC Executive. The ALSPAC data management plan describes in detail the policy

regarding data sharing, which is through a system of managed open access. Full instructions for applying for data access can be found here: http://www.bristol.ac.uk/alspac/researchers/access/. The ALSPAC study website contains details of all the data that are available (http://www.bristol.ac.uk/alspac/researchers/our-data/).

Research involving human participants, their data, or biological material

Policy information about studies with human participants or human data. See also policy information about sex, gender (identity/presentation), and sexual orientation and race, ethnicity and racism.

Reporting on sex and gender	Sex as a biological variable was collected, used in the analysis and reported.
Reporting on race, ethnicity, or other socially relevant groupings	Majority of the population over 95% are White from Bristol area of the UK. Thus there were no ethnicity stratified results.
Population characteristics	See above and the recruitment section below.
Recruitment	Data were from the Avon Longitudinal Study of Parents and Children (ALSPAC) birth cohort, which investigates factors that influence childhood development and growth. Pregnant women resident in Avon, UK with expected dates of delivery between 1st April 1991 and 31st December 1992 were invited to take part in the study. 20,248 pregnancies have been identified as being eligible and the initial number of pregnancies enrolled was 14,541. Of the initial pregnancies, there was a total of 14,676 foetuses, resulting in 14,062 live births and 13,988 children who were alive at 1 year of age. When the oldest children were approximately 7 years of age, an attempt was made to bolster the initial sample with eligible cases who had failed to join the study originally. As a result, when considering variables collected from the age of seven onwards (and potentially abstracted from obstetric notes) there are data available for more than the 14,541 pregnancies mentioned above. The number of new pregnancies not in the initial sample (known as Phase I enrolment) that are currently represented in the released data and reflecting enrolment status at the age of 24 is 906, resulting in an additional 913 children being enrolled (456, 262 and 195 recruited during Phases II, III and IV respectively). The total sample size for analyses using any data collected after the age of seven is therefore 15,447 pregnancies, resulting in 15,658 foetuses. Of these 14,901 children were alive at 1 year of age. Regular clinic visits of the children commenced at 7 years of age and are still ongoing into adulthood.
Ethics oversight	Ethical approval for the study was obtained from the ALSPAC Ethics and Law Committee and the Local Research Ethics Committees as listed below.<br>11 Year Clinic:<br>• Southmead (North Bristol Trust): 137/02: ALSPAC – Focus at 11+. (17th April 2003)<br>• United Bristol Healthcare Trust: E5691 ALSPAC Focus @ 11+. [Reciprocal] (29th May 2003)<br>• Frenchay (North Bristol Trust): 2002/110 Avon Longitudinal Study of Parents and Children (ALSPAC) Assessments at Age Eleven Plus. (23rd June 2003)<br>• Weston Area Health Trust: E325 (R) – 137/02 Avon Longitudinal Study of Parents and Children (ALSPAC) Assessments at Age Eleven Plus. (28th February 2003)<br><br>15 Year Clinic:<br>• Central & South Bristol Research Ethics Committee (UBHT): 06/Q2006/53 Avon Longitudinal Study of Parents and Children (ALSPAC), Hands on Assessments: Teen Focus 3 (Focus 15+). (7th August 2006) (Confirmed 15th September 2006)<br><br>17 Year clinic:<br>• North Somerset & South Bristol Research Ethics Committee: 08/H0106/9 Avon Longitudinal Study of Parents and Children (ALSPAC), Hands on Assessments: Teen Focus 4 (Focus 17+) (18th November 2008)<br><br>24 Year Clinic:<br>• National Research Ethics Service Committee South West – Frenchay: 14/SW/1173 ALSPAC Focus at 24+ (24th February 2015, confirmed 20th March 2015)<br><br>Informed consent for the use of data collected via questionnaires and clinics was obtained from participants following the recommendations of the ALSPAC Ethics and Law Committee at the time. Consent for biological samples has been collected in accordance with the Human Tissue Act (2004).

Note that full information on the approval of the study protocol must also be provided in the manuscript.

Field-specific reporting

Please select the one below that is the best fit for your research. If you are not sure, read the appropriate sections before making your selection.

☒ Life sciences ☐ Behavioural & social sciences ☐ Ecological, evolutionary & environmental sciences

For a reference copy of the document with all sections, see nature.com/documents/nr-reporting-summary-flat.pdf

Life sciences study design

All studies must disclose on these points even when the disclosure is negative.

Sample size	This study population was drawn from a birth cohort as detailed below. Pregnant women resident in Avon, UK with expected dates of delivery

	between 1st April 1991 and 31st December 1992 were invited to take part in the study. 20,248 pregnancies have been identified as being eligible and the initial number of pregnancies enrolled was 14,541. Of the initial pregnancies, there was a total of 14,676 foetuses, resulting in 14,062 live births and 13,988 children who were alive at 1 year of age. When the oldest children were approximately 7 years of age, an attempt was made to bolster the initial sample with eligible cases who had failed to join the study originally. As a result, when considering variables collected from the age of seven onwards (and potentially abstracted from obstetric notes) there are data available for more than the 14,541 pregnancies mentioned above. The number of new pregnancies not in the initial sample (known as Phase I enrolment) that are currently represented in the released data and reflecting enrolment status at the age of 24 is 906, resulting in an additional 913 children being enrolled (456, 262 and 195 recruited during Phases II, III and IV respectively). The total sample size for analyses using any data collected after the age of seven is therefore 15,447 pregnancies, resulting in 15,658 foetuses. Of these 14,901 children were alive at 1 year of age.<br>At age 11years only 7159 children attended the clinic visit, at age 15 years only 5509 adolescents attended the clinic visit and at age 24 years only 4026 attended the clinic visit. All participants who had at least one assessment of movement behaviour and body composition during the 13 year follow up were included. n = 6059 children aged 11 years at baseline.<br>Sample size calculation for this cohort was already published Golding et al. ALSPAC - The Avon Longitudinal Study of Parents and Children I. Study methodology. Paediatr Perinat Epidemiol. 2001;15(1):74-87. doi:10.1046/j.1365-3016.2001.00325.x<br>Analyses involving 50% of a sample of 10,000 ALSPAC children at 0.8 statistical power, 0.05 alpha, and 2-sided p-value would show a minimum detectable effect size of 0.048 standard deviations if they had relevant exposure for a normally distributed quantitative variable.
Data exclusions	All participants who had at least one assessment of movement behaviour and body composition during the 13 year follow up were included. n = 6059 children aged 11 years at baseline. Only 303 children who had at least one time-point measure of movement behaviour but do not have data on dual energy Xray absoprtiometry measured body composition were excluded.
Replication	Results were replicated in the same population using different inclusion criteria resulting in different sample sizes. This replication was successful. Unfortunately, we could not replicate the study in a different population as our search attempt for other cohorts with repeated measures of accelerometer-based movement behaviour and dual energy Xray absorptimetry body composition from childhood to young adulthood was unsuccessful due to lack of availability of this data across the globe as of June 2023.
Randomization	Not applicable due to prospective study design of the birth cohort. Not a randomized controlled trial.
Blinding	Not applicable due to prospective study design of the birth cohort. Not a randomized controlled trial.

Reporting for specific materials, systems and methods

We require information from authors about some types of materials, experimental systems and methods used in many studies. Here, indicate whether each material, system or method listed is relevant to your study. If you are not sure if a list item applies to your research, read the appropriate section before selecting a response.

Materials & experimental systems

n/a	Involved in the study	
☒	☐	Antibodies
☒	☐	Eukaryotic cell lines
☒	☐	Palaeontology and archaeology
☒	☐	Animals and other organisms
☒	☐	Clinical data
☒	☐	Dual use research of concern
☒	☐	Plants

Methods

n/a	Involved in the study	
☒	☐	ChIP-seq
☒	☐	Flow cytometry
☒	☐	MRI-based neuroimaging
